# Author Correction: SARS-CoV-2 uses metabotropic glutamate receptor subtype 2 as an internalization factor to infect cells

**DOI:** 10.1038/s41421-021-00365-z

**Published:** 2021-12-27

**Authors:** Jinliang Wang, Guan Yang, Xinxin Wang, Zhiyuan Wen, Lei Shuai, Jie Luo, Chong Wang, Ziruo Sun, Renqiang Liu, Jinying Ge, Xijun He, Ronghong Hua, Xijun Wang, Xiao Yang, Weiye Chen, Gongxun Zhong, Zhigao Bu

**Affiliations:** 1 State Key Laboratory of Veterinary Biotechnology, Harbin Veterinary Research Institute, Chinese Academy of Agricultural Sciences, Harbin, Heilongjiang China; 2 State Key Laboratory of Proteomics, Beijing Proteome Research Center, National Center for Protein Sciences (Beijing), Beijing Institute of Lifeomics, Beijing, China

**Keywords:** Mechanisms of disease, Molecular biology

Correction to: *Cell Discovery* (2021) 7:119

10.1038/s41421-021-00357-z published online 14 December 2021

In the original publication of this article^1^, we made some mistakes in Fig. 4c and d. The layer of nucleus was missing in the images of CK5 and CK8 of Fig. 4c, and the layer of Ace2 was shifted in the image of Ace2 of Fig. 4d. The correctly labelled Fig. 4c and d are displayed as below. This correction does not affect the results or the conclusion of this work.

**Figure:**
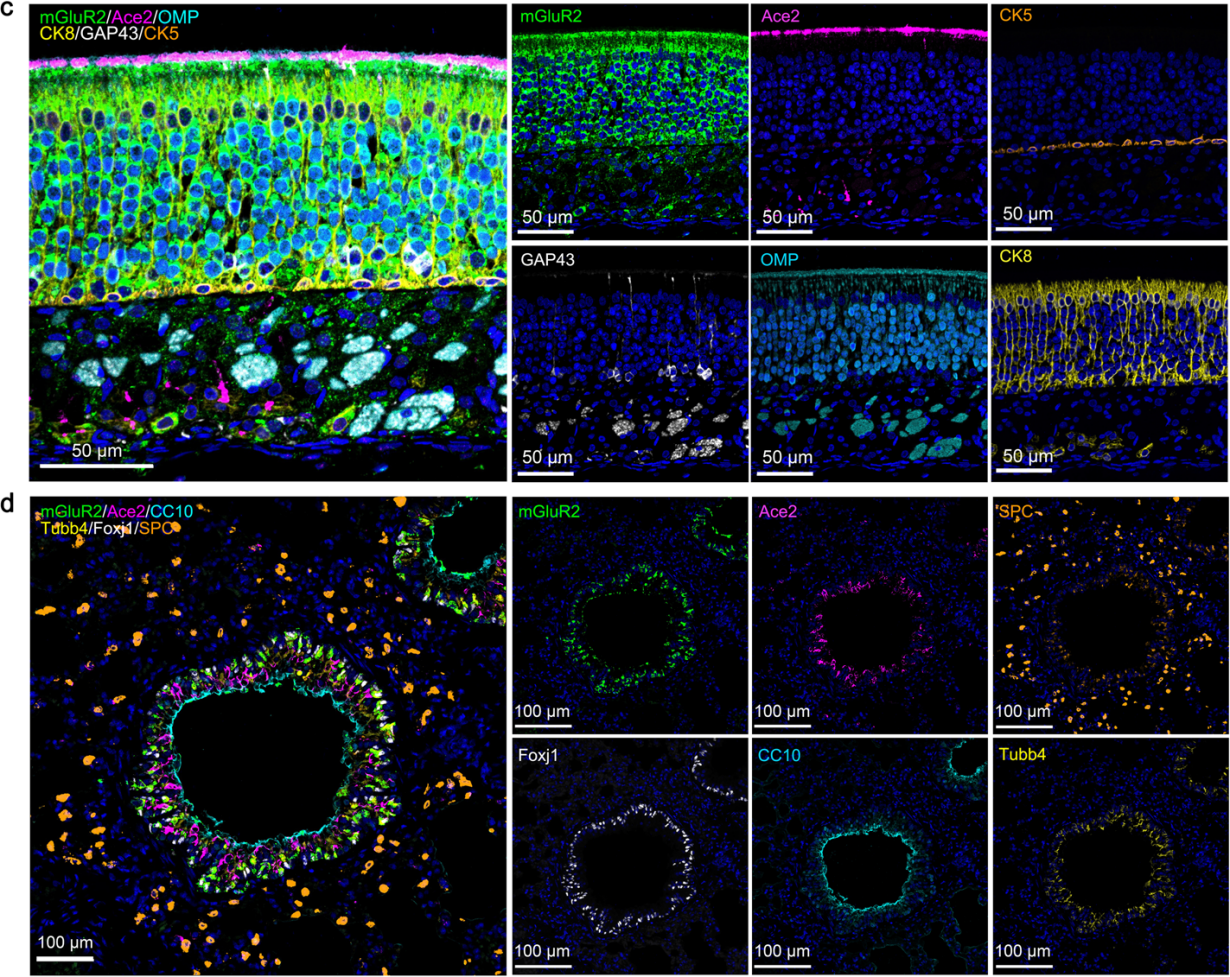

## References

[1] Wang J (2021). SARS-CoV-2 uses metabotropic glutamate receptor subtype 2 as an internalization factor to infect cells. Cell Discov..

